# Classical Food Fermentations as Modern Biotechnological Platforms: Alcoholic, Acetic, Butyric, Lactic and Propionic Pathways and Applications

**DOI:** 10.3390/molecules31020333

**Published:** 2026-01-19

**Authors:** Anna Rymuszka, Wiktoria Gorczynska

**Affiliations:** 1 Department of Physiology and Toxicology, Faculty of Medicine, The John Paul II Catholic University of Lublin, Konstantynów Str. 1i, 20-708 Lublin, Poland; 2 Independent Researcher, 20-950 Lublin, Poland; gorczynska.wikt@gmail.com

**Keywords:** alcoholic fermentation, acetic acid bacteria, butyric acid, lactic acid bacteria, propionic acid, fermented foods, bioprocessing, bio-based chemicals, functional foods

## Abstract

Fermentation remains central to food manufacturing and to the bio-based production of organic acids, solvents, and functional metabolites. This review integrates the biochemical pathways, key microorganisms, and application space of five major industrial fermentations—alcoholic, acetic, butyric, lactic, and propionic. We summarize the principal metabolic routes (EMP/ED glycolysis; oxidative ethanol metabolism; butyrate-forming pathways; and the Wood–Werkman, acrylate, and 1,2-propanediol routes to propionate) and relate them to the dominant microbial groups involved, including yeasts, acetic acid bacteria, lactic acid bacteria, clostridia, and propionibacteria. We highlight how the resulting metabolite spectra—ethanol, acetic acid, butyrate, lactate, propionate, and associated secondary metabolites—underpin product quality and safety in fermented foods and beverages and enable the industrial synthesis of platform chemicals, polymers, and biofuels. Finally, we discuss current challenges and opportunities for sustainable fermentation, including waste stream valorization, process intensification, and the integration of systems biology and metabolic engineering within circular economy frameworks.

## 1. Introduction

Fermentation is one of the oldest biotechnological processes, in which microorganisms, through their metabolic activity, transform plant- or animal-based raw materials into products with modified physicochemical and sensory properties [1,2,3,4]. From a biochemical standpoint, fermentation is a metabolic process in which energy is generated from organic compounds in the absence of an exogenous electron acceptor [5]. During fermentation, microorganisms catabolize carbohydrates into alcohols, carbon dioxide and/or organic acids, while simultaneously utilizing nutrients present in raw materials, such as carbon, nitrogen, vitamins and trace elements, for growth and reproduction [1]. As a result of microbial enzymatic activity, complex organic molecules undergo chemical and physical transformations and are converted into simpler, bioactive, functional and nutritionally beneficial compounds [6]. The metabolic reactions occurring during fermentation not only alter food composition but also enhance its nutritional, sensory and functional characteristics [7]. This biochemical capacity has been exploited empirically since antiquity, long before the microbial basis of fermentation was understood.

The origins of fermentation can be traced back to early human societies, with archaeological evidence indicating its use as early as the Late Epipaleolithic period. Residues of fermented cereal-based beverages discovered at Raqefet Cave in present-day Israel provide the earliest known proof of deliberate fermentation, highlighting its role in early food processing practices. Over subsequent millennia, fermentation became a fundamental method of food production and preservation across ancient civilizations, including Mesopotamia, Egypt, and China, where it was used for the manufacture of bread, beer, wine, and other fermented products. Initially, fermentation relied on spontaneous processes driven by indigenous microbiota associated with raw materials and the environment. Through empirical observation, humans gradually learned to manipulate fermentation conditions to improve product quality and stability. The biological basis of fermentation remained unknown until the 19th century, when Louis Pasteur demonstrated its microbial nature. This discovery enabled intentional strain selection and process optimization, ultimately laying the foundation for modern industrial biotechnology [8,9,10]. Today, fermentation is increasingly integrated with systems biology and metabolic engineering to improve productivity, robustness, and sustainability of bioprocesses. In practice, these tools enable the identification of metabolic bottlenecks and stress-response limitations and support rational strain and process design to improve yields, robustness, and substrate utilization.

Accordingly, modern fermentation practice is often distinguished by the degree of microbial and process control applied during production. Two primary approaches to food fermentation are recognized. The first is spontaneous fermentation, driven by autochthonous microorganisms naturally present in raw materials or the processing environment [3,11]. Due to the undefined composition and dynamics of the microbial communities involved, spontaneous fermentation requires strict monitoring and stabilization [6]. The second approach involves the use of starter cultures, which dominate contemporary industrial practice. Starter cultures consist of selected and well-characterized strains of bacteria, yeasts or molds that initiate and guide fermentation in a controlled, reproducible manner, suppressing undesirable microorganisms and enhancing food safety [12]. These cultures accelerate fermentation, convert available carbohydrates into alcohols and organic acids that act as natural preservatives, and impart desirable sensory attributes. Their application standardizes product quality, extends shelf life and reduces technological variability. In addition, many strains exhibit antagonistic activity toward pathogens, supporting microbial safety. For these reasons, ongoing research focuses on isolating and characterizing strains with optimal technological and probiotic potential for use as functional starter cultures [6,12].

While spontaneous fermentations are increasingly valued for their contribution to product diversity, regional identity, and complex sensory profiles, their inherent microbial variability limits their applicability in large-scale industrial production. In contrast, defined starter cultures enable reproducible fermentation kinetics, improved safety through rapid acidification or ethanol production, and consistent product quality. Consequently, modern food fermentation often combines traditional practices with controlled starter cultures or selected mixed consortia to balance authenticity with industrial reliability, particularly in processes requiring scalability and compliance with safety standards [9]. 

Fermentation technologies therefore continue to play a key role in the food sector, improving preservation, ensuring microbiological safety, enhancing sensory quality, and enabling the transformation of perishable raw materials into stable, shelf-stable, and nutritionally valuable products [4]. Because these pathways are metabolically connected and often occur sequentially or in mixed-culture systems, a comparative overview is essential to capture fermentation as an integrated biotechnological platform rather than isolated processes. Depending on the substrates and microorganisms involved, five major types of fermentation can be distinguished: alcoholic, acetic, butyric, lactic and propionic.

The aim of this review is to provide a comprehensive and integrative overview of the biochemical pathways, microbial ecology, and industrial applications of these five major fermentation types. The article synthesizes current knowledge on the metabolic mechanisms underlying each process, the microorganisms involved, their technological roles and their relevance in the food, pharmaceutical and biotechnological industries. Additionally, the review highlights recent advances, emerging trends and future prospects in fermentation-based bioprocessing, with particular emphasis on sustainability, functional food development and the valorization of biological raw materials. Key barriers to sustainable implementation include heterogeneous and seasonally variable waste streams, inhibitory compounds in low-cost substrates, and the need for robust strains and scalable downstream processing. In addition to summarizing classical pathways and applications, this review addresses cross-cutting industrial challenges such as process scale-up, yield optimization, and economic feasibility, and discusses emerging trends in sustainable and data-driven fermentation technologies. In the following sections, each fermentation type is discussed within a consistent framework, progressing from core biochemical reactions to the principal microorganisms involved and, finally, to representative industrial applications. By jointly analyzing five archetypal fermentation types rather than a single product, this review highlights fermentation as a modular and interconnected biotechnological platform, in which distinct pathways collectively support food production, platform chemicals, biofuels, and functional biomaterials.

## 2. Alcoholic Fermentation

### 2.1. Biochemical Basis

Alcoholic (ethanolic) fermentation is an anaerobic biochemical process in which sugars such as glucose, fructose, and sucrose are converted into ethyl alcohol (ethanol) and carbon dioxide [1,3,8,10,13,14]. The process begins with glycolysis—a series of enzyme-catalyzed reactions that degrade one molecule of glucose into two molecules of pyruvate, accompanied by the formation of two molecules of ATP and two molecules of NADH. In the next step, pyruvate undergoes decarboxylation catalyzed by pyruvate decarboxylase, producing acetaldehyde and releasing carbon dioxide. Subsequently, acetaldehyde is reduced to ethanol in a reaction catalyzed by alcohol dehydrogenase, during which NADH is oxidized back to NAD^+^. The regeneration of NAD^+^ enables the continuation of glycolysis and energy production under anaerobic conditions [7,8,13,15,16,17]. The overall reaction can be expressed as follows:

C_6_H_12_O_6_ → 2 C_2_H_5_OH + 2 CO_2_

While the overall stoichiometry is conserved, ethanol yield, productivity, and stress tolerance depend strongly on the microorganism and the glycolytic route used, as outlined below.

### 2.2. Microorganisms and Metabolic Pathways

Alcoholic fermentation is carried out primarily by yeasts and, to a lesser extent, by certain bacterial species [18,19,20]. The most commonly employed microorganism is *Saccharomyces cerevisiae*, which ferments glucose via the classical glycolytic pathway known as the Embden–Meyerhof–Parnas (EMP) pathway. In this route, each molecule of glucose is converted into two molecules of pyruvate and subsequently—according to the mechanism described above—into ethanol and carbon dioxide [15,20,21,22].

Certain bacteria, such as *Zymomonas mobilis*, utilize an alternative sugar catabolic pathway—the Entner–Doudoroff (ED) pathway. Similarly to the EMP pathway, it leads to the formation of pyruvate but is characterized by a lower energetic yield, producing only one ATP molecule per molecule of glucose, and by reduced biomass formation, thereby channeling a higher proportion of sugar toward ethanol production [15,18,19,21].

**Figure 1:**
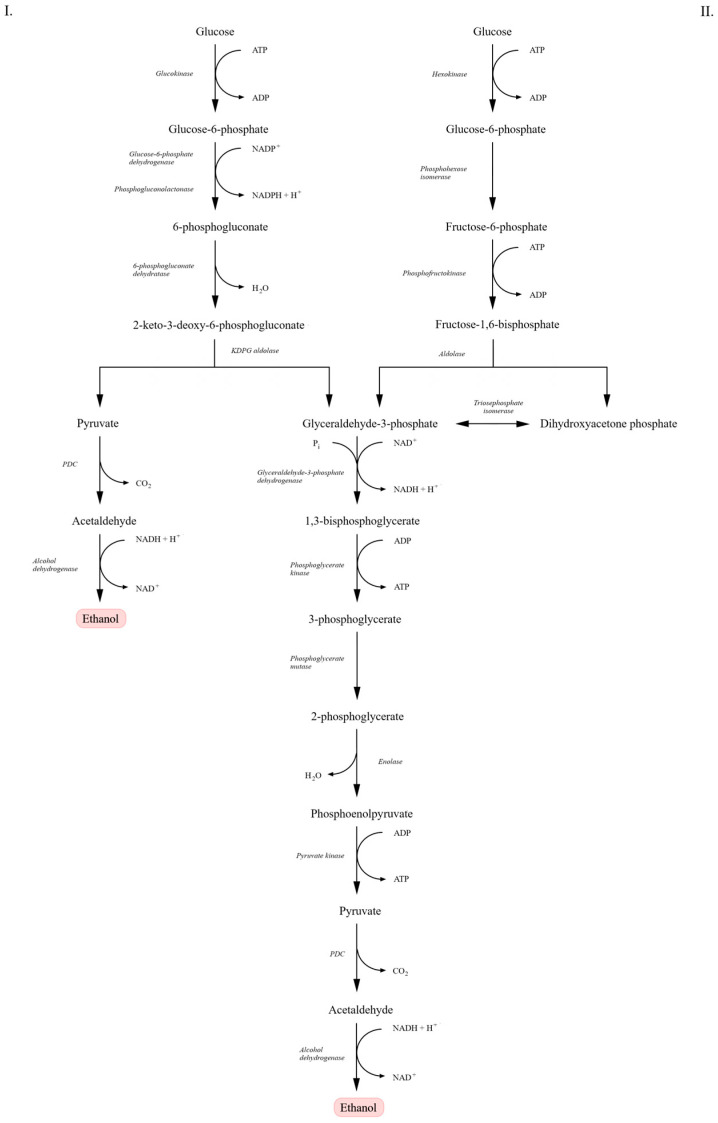
Comparison of the Entner–Doudoroff (ED) and Embden–Meyerhof–Parnas (EMP) pathways leading to alcoholic fermentation. In the ED pathway (**I**), glucose is converted through glucose-6-phosphate, 6-phosphogluconate, and 2-keto-3-deoxy-6-phosphogluconate (KDPG), yielding pyruvate and glyceraldehyde-3-phosphate. In the EMP pathway (**II**), glucose is metabolized through glucose-6-phosphate, fructose-1,6-bisphosphate, and glyceraldehyde-3-phosphate. In both pathways, pyruvate is decarboxylated to acetaldehyde by pyruvate decarboxylase (PDC), followed by reduction to ethanol by alcohol dehydrogenase (ADH). Scheme adapted from [21].

As illustrated in Figure 1, the ED pathway differs from the classical EMP route in both reaction sequence and enzymatic machinery. In the EMP pathway, glucose is metabolized to pyruvate with the net production of two ATP and two NADH molecules. A key enzyme, fructose-1,6-bisphosphate aldolase, cleaves fructose-1,6-bisphosphate into glyceraldehyde-3-phosphate and dihydroxyacetone phosphate. In contrast, in the ED pathway, glucose-6-phosphate is converted into 6-phosphogluconate and subsequently into 2-keto-3-deoxy-6-phosphogluconate (KDPG), which is cleaved by KDPG aldolase directly into pyruvate and glyceraldehyde-3-phosphate—yielding lower energy output [21,23,24]. Despite differences in the early steps of glucose metabolism, the downstream reactions—conversion of glyceraldehyde-3-phosphate to pyruvate—proceed identically in both pathways, involving the same enzymes and resulting in the same end products. In each case, pyruvate is further converted to ethanol and carbon dioxide [15,22].

Although the EMP pathway yields more ATP per mole of glucose than the ED pathway, this higher energy conservation in *S. cerevisiae* is generally associated with increased biomass formation and stress tolerance rather than maximal ethanol yield [16,23,25]. In contrast, the lower ATP yield of the ED pathway in *Z. mobilis* can limit biomass synthesis and direct a higher fraction of carbon toward ethanol production, resulting in higher specific ethanol productivity despite lower energetic efficiency [18,23,24].

The choice of microorganism and metabolic route depends on physiology, ethanol yield, fermentation rate, substrate spectrum and tolerance to inhibitory compounds. Yeasts such as *S. cerevisiae* are valued for their high ethanol tolerance, robustness under industrial conditions, and capacity to withstand osmotic, thermal, and inhibitory stresses. These features, together with its genetic stability, extensive industrial know-how, and availability of advanced molecular tools, explain why *S. cerevisiae* remains the dominant microorganism in industrial alcoholic fermentation [25,26]. In contrast, *Z. mobilis* exhibits high ethanol productivity, rapid fermentation rates, and low biomass formation due to the lower ATP yield of the ED pathway, making strain selection strongly dependent on the specific requirements of a given bioprocess [19]. These metabolic features directly translate into technological performance, which explains why specific strains are preferred for beverage, baking, and biofuel applications.

In recent years, alcoholic fermentation has become a major target of metabolic engineering and synthetic biology aimed at improving ethanol yield, productivity, and stress tolerance under industrial conditions [25,27,28]. In *S. cerevisiae*, engineering strategies have focused on redirecting carbon flux toward ethanol by minimizing by-product formation (e.g., glycerol and organic acids), enhancing cofactor balance, and increasing tolerance to ethanol, temperature, and osmotic stress [16,25,29]. Genome-scale metabolic models and CRISPR/Cas-based genome editing have enabled systematic modification of glycolytic regulation, redox metabolism, and membrane composition, resulting in strains with improved robustness and fermentation performance [25,30,31,32].

In parallel, *Z. mobilis* has been extensively engineered to expand its substrate spectrum beyond glucose to pentoses such as xylose and arabinose, facilitating the efficient conversion of lignocellulosic hydrolysates into ethanol [24,33]. Synthetic biology approaches, including pathway modularization, dynamic gene regulation, and adaptive laboratory evolution, are increasingly integrated with process engineering to optimize large-scale bioethanol production and reduce overall process costs [18,34,35]. These advances highlight the transition of alcoholic fermentation from a classical bioprocess to a data-driven and design-oriented platform for sustainable biofuel and biochemical production [25,27].

### 2.3. Industrial Applications

Alcoholic fermentation is a biotechnological process with a wide range of industrial applications, encompassing the food, biotechnological, chemical, and energy sectors (Table 1). Its ability to convert carbohydrates into ethanol and other bioactive metabolites makes it a cornerstone of both traditional and modern bioprocessing technologies [9,26].

This type of fermentation represents one of the oldest and most fundamental biotechnological processes applied in the food industry [7,36,37,38]. The classical examples include the production of wine, beer, cider, mead, and distilled beverages such as whisky, rum, and vodka. In these processes, yeasts—primarily *S. cerevisiae*—convert sugars present in raw materials such as grape must, barley mash, apple juice, or honey solutions into ethanol and carbon dioxide. For distilled beverages, fermentation serves as a preliminary stage before distillation, which increases ethanol concentration. In addition to ethanol, numerous aromatic compounds, such as esters, aldehydes, and higher alcohols are generated, contributing to the sensory complexity and quality of the final beverages. For example, ethyl esters (e.g., ethyl acetate, ethyl hexanoate) impart fruity and floral notes, higher alcohols contribute complexity and mouthfeel, and aldehydes influence freshness and balance, collectively shaping the characteristic sensory profiles of fermented beverages [8,39,40,41,42].

Alcoholic fermentation also plays an important role in the production of low-alcohol fermented beverages, including water kefir, kombucha, kvass, and ginger beer. In the initial stage of these processes, yeasts carry out alcoholic fermentation, resulting in small amounts of ethanol, which is subsequently oxidized to acetic acid by acetic acid bacteria. The final product therefore contains only trace amounts of alcohol, and its sensory and nutritional value arises from fermentation metabolites and microorganisms with potential probiotic properties [9,37].

Beyond beverage production, alcoholic fermentation has significant applications in biotechnology for the biosynthesis of natural flavors, enzymes, and aromatic components. During fermentation, yeasts generate ethyl esters, aldehydes, and ketones that impart characteristic aroma profiles and are widely utilized in the dairy, bakery, and confectionery industries [16,36,43,44]. In bakery processes, *S. cerevisiae* metabolizes sugars in flour into ethanol and carbon dioxide, leading to dough leavening and crumb formation. Although ethanol evaporates during baking, intermediate metabolites formed during fermentation contribute to the desirable flavor and aroma of baked goods [45,46].

In the chemical and pharmaceutical sectors, ethanol obtained via yeast fermentation is a valuable raw material and universal solvent. It serves as a substrate for the synthesis of various organic compounds such as ethylene, ethyl acetate, diethyl ether, acetic acid, glycerol, and organic acids (e.g., lactic and succinic), which are employed in the production of pharmaceuticals, cosmetics, plastics, solvents, and preservatives [7,29,47,48]. In pharmacy, ethanol functions as a carrier for active substances, a preservative, a disinfectant, and a technological agent in extraction and stabilization processes of bioactive compounds [36,43,49].

In modern bioenergy technologies, alcoholic fermentation is used for the production of bioethanol—a renewable and low-emission fuel that serves as a sustainable alternative to fossil fuels [14,19,25,48,50]. Fermentation-derived ethanol also plays an important role in green chemistry, acting as a feedstock for the synthesis of bioplastics, bioesters, and other bio-based fuel components, thus supporting the development of a circular and sustainable economy [9,19].

Alcoholic fermentation further holds cultural and historical importance. In many regions worldwide, it forms part of culinary heritage and serves as the basis for traditional beverages such as Japanese sake, Eastern European kvass, South American chicha, and Asian plant-based drinks such as pulque, tuba, and toddy. These processes, often carried out by wild yeasts or naturally occurring microbial consortia, give products unique sensory and cultural characteristics [36,51,52,53,54].

**Table 1:** Representative industrial applications of alcoholic fermentation in the food, chemical and energy sectors.

Application Area	Example Products	Main Microorganisms	Fermentation Products/Effects	Technological Significance	Typical Product Concentration	Representative References
Alcoholic beverages	Wine, beer, cider, mead, spirits (whisky, rum, vodka)	*Saccharomyces cerevisiae*, *S. bayanus*, *Schizosaccharomyces pombe*	Ethanol, CO_2_, esters, higher alcohols, aldehydes	Alcohol production; development of aroma and flavor; natural preservation	Beer: 3–8% (*v*/*v*); Wine: 10–15% (*v*/*v*); Spirits (after distillation): >40% (*v*/*v*)	[8,26,36,46,55,56,57]
Low-alcohol fermented drinks	Kombucha, kvass, water kefir, ginger beer	*Saccharomyces*, *Zygosaccharomyces*, *Acetobacter*, *Lactobacillus*	Ethanol → acetic acid, CO_2_, organic acids	Light carbonation; improved flavor and probiotic potential	<1% (*v*/*v*) ethanol	[9,12,58,59,60,61,62,63,64,65,66]
Baking and confectionery	Bread, rolls, yeast doughs	*Saccharomyces cerevisiae*	CO_2_, volatile ethanol, esters	Dough leavening; improved texture, flavor, and aroma	Ethanol transient; CO_2_ for leavening (ethanol evaporates during baking)	[14,36,43,46,67,68,69]
Flavor and aroma production	Natural flavors, fruit and dairy aroma compounds	*Saccharomyces*, *Kluyveromyces*, *Torulaspora* spp.	Esters, aldehydes, ketones, higher alcohols	Development of natural flavoring ingredients for food and beverages	Trace levels (mg·L^−1^ range)	[8,36,40,43,57,70,71,72,73]
Pharmaceutical and chemical industry	Solvents, acetic acid, ethyl acetate, diethyl ether, glycerol	*S. cerevisiae*, *Candida*, *Kluyveromyces* spp.	Ethanol, organic acids, secondary metabolites	Production of solvents, preservatives, and pharmaceutical intermediates	Ethanol typically >95% (*v*/*v*) after purification	[7,14,25,36,47,74]
Bioenergy sector	Bioethanol fuel, bioplastics, bioesters	*S. cerevisiae*, *Zymomonas mobilis*	Ethanol, CO_2_	Renewable energy generation; sustainable fuel and biocomponent production	80–120 g·L^−1^ ethanol	[14,25,33,36,47,74,75]
Cultural and traditional fermentation	Sake, chicha, kvass, pulque, toddy, tuba	Wild yeasts, mixed consortia (*Saccharomyces*, *Lactobacillus*)	Ethanol, CO_2_, esters, organic acids	Preservation of traditional food heritage; unique sensory profiles	Typically 2–8% (*v*/*v*) ethanol	[26,36,39,46,53,54,76,77,78,79]

Typical ethanol concentrations range from approximately 3–8% (*v*/*v*) in beer, 10–15% (*v*/*v*) in wine, <1% (*v*/*v*) in low-alcohol fermented beverages, and >80–100 g·L^−1^ in industrial bioethanol fermentations, reflecting differences in microbial physiology, pathway utilization (EMP versus ED), and process objectives [9,18,24,25,26,41,64].

## 3. Acetic Fermentation

Following alcoholic fermentation, acetic fermentation represents a distinct yet functionally connected process in which ethanol is further oxidized by specialized bacteria under aerobic conditions, linking primary sugar catabolism with organic acid production.

### 3.1. Biochemical Pathway and Key Microorganisms

The production of acetic acid represents a complex, two-step metabolic pathway involving different groups of microorganisms and specialized enzymatic systems. The first stage comprises alcoholic fermentation, occurring under anaerobic conditions, during which simple sugars, such as glucose are converted into ethanol and carbon dioxide by the action of yeasts, mainly of the genus *Saccharomyces*. The subsequent stage, proceeding in the presence of oxygen, involves acetic acid bacteria (AAB) that oxidize ethanol to acetic acid and water [80], according to the reaction:

$$C_{2}H_{5}\mathrm{OH}+O_{2}\to {\mathrm{CH}}_{3}\mathrm{COOH}+H_{2}O$$

Both stages require microorganisms with high adaptive capacity, enabling their activity under variable and often unfavorable environmental conditions. During alcoholic fermentation, the use of yeast strains with high alcohol tolerance is essential to ensure efficient ethanol production. In the acetic acid fermentation phase, the selection of bacterial strains capable of withstanding both high ethanol concentrations and the accumulation of acetic acid is crucial, as this process demands dual tolerance to both compounds [81].

The most important acetic acid–producing bacteria used in biotechnological vinegar production belong to the genera *Acetobacter*, *Gluconacetobacter*, *Gluconobacter*, and *Komagataeibacter*. These strains exhibit high efficiency in ethanol oxidation to acetic acid and substantial tolerance to product accumulation in the fermentation environment [81,82].

The mechanism of acetic acid fermentation is based on a specialized membrane-bound enzymatic system located in the cytoplasmic membrane of acetic acid bacteria. Two key dehydrogenases are involved in this process: the pyrroloquinoline quinone (PQQ)-dependent alcohol dehydrogenase (ADH) and the acetaldehyde dehydrogenase (ALDH). The first enzyme catalyzes the oxidation of ethanol to acetaldehyde, while the second converts acetaldehyde into acetic acid. Both dehydrogenases are membrane-bound and oriented toward the periplasmic space, allowing direct oxidation of substrates located outside the cytoplasm and the release of products into the extracellular medium [83,84,85,86].

Electrons released during ethanol and acetaldehyde oxidation are transferred from PQQ to ubiquinone (UQ), which acts as a mobile electron carrier. Reduced ubiquinone (ubiquinol, UQH_2_) donates electrons to ubiquinol oxidase (UOX), which transfers them to molecular oxygen, the terminal electron acceptor. This reaction yields water, while proton translocation across the membrane generates a proton motive force that drives ATP synthesis via ATP synthase [83,84,85,86].

**Figure 2:**
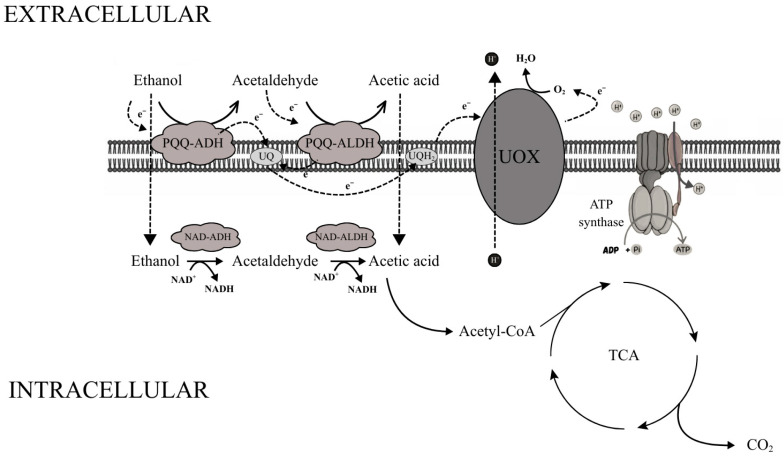
Schematic representation of the membrane-bound and cytosolic respiratory chains involved in ethanol oxidation in acetic acid bacteria. In this system, periplasmic PQQ-dependent dehydrogenases catalyze the oxidation of ethanol and acetaldehyde, transferring electrons through ubiquinone to the terminal ubiquinol oxidase (UOX), which drives proton translocation and ATP formation. Intracellularly, acetate is further converted to acetyl-CoA and enters the tricarboxylic acid (TCA) cycle. Scheme adapted from [83].

In addition to the membrane-bound system, AAB possess a cytoplasmic dehydrogenase complex using NAD^+^/NADP^+^ as cofactors. NAD-dependent alcohol dehydrogenase (NAD-ADH) and NADP-dependent acetaldehyde dehydrogenase (NADP-ALDH) oxidize ethanol that diffuses into the cytoplasm to acetic acid; the latter can be converted into acetyl-CoA, which enters the tricarboxylic acid (TCA) cycle and is fully oxidized to CO_2_ and water (Figure 2) [23,83,87]. Thus, the coupling of periplasmic ethanol oxidation to proton motive force generation enables efficient ATP synthesis under high ethanol and acidic conditions, while the combined ethanol and acid tolerance of yeasts and acetic acid bacteria directly underpins their robustness and efficiency in industrial acetic fermentation systems [83,87,88,89].

Under conditions relevant for acetic acid production, the membrane-bound PQQ-ADH and ALDH dominate, whereas cytoplasmic NAD-ADH and NADP-ALDH are largely inhibited. High ethanol concentrations favor the membrane pathway. As ethanol is depleted, cytoplasmic dehydrogenase activity increases and the contribution of the membrane system declines. This dynamic regulation allows AAB to optimize energy conservation and adapt to shifting environmental conditions [83,85,87,90]. Together, these metabolic adaptations explain why acetic acid bacteria are uniquely suited for industrial vinegar production and other acetic acid-based bioprocesses.

Moreover, with traditional strain selection, acetic acid bacteria have increasingly become targets of metabolic engineering and systems-level optimization aimed at improving acetic acid productivity, acid tolerance, and process robustness [83,87,91,92]. Recent studies have focused on enhancing ethanol and acetic acid tolerance through modification of membrane lipid composition, overexpression of stress response regulators, and optimization of respiratory chain components involved in periplasmic ethanol oxidation [83,85,90]. Advances in genome sequencing and comparative genomics of *Acetobacter*, *Komagataeibacter*, and *Gluconacetobacter* species have enabled the identification of key genes associated with acid resistance, oxidative stress defense, and efficient energy conservation [93].

Emerging synthetic biology approaches, including targeted gene deletions, controlled overexpression of PQQ-dependent dehydrogenases, and adaptive laboratory evolution, have further improved strain performance under high-acidity and high-ethanol conditions relevant to industrial vinegar production [94]. Coupled with bioprocess intensification strategies and real-time monitoring of dissolved oxygen and redox status, these developments highlight the transition of acetic fermentation from a largely empirical process to a rationally engineered biotechnological platform [84,95].

### 3.2. Industrial Applications

Building on the biochemical mechanisms described above, acetic fermentation has been widely exploited in both food and non-food industries due to its ability to generate acetic acid and related metabolites with preservative, sensory, and functional properties. While vinegar production remains the most recognized application, acetic fermentation should be viewed as a platform bioprocess supporting multiple industrial sectors.

Acetic fermentation plays a crucial role across the food industry, extending beyond conventional vinegar production. The most widespread application is the manufacture of edible vinegars, which are aqueous acetic acid solutions typically containing 4–15% (*w*/*v*) acetic acid. Depending on the raw material and technology used, spirit vinegar (from ethanol), wine vinegar, apple cider vinegar, and rice vinegar can be distinguished, each with characteristic sensory and nutritional properties [61,82]. Acetic acid content determines preservative, antimicrobial and organoleptic attributes. Vinegar functions as both a condiment and a natural preservative due to its ability to lower pH and inhibit undesirable microflora. Commercial vinegar production commonly involves a two-stage fermentation: alcoholic fermentation by yeasts (e.g., *S. cerevisiae*), followed by acetic oxidation by AAB [36,54]. A summary of the major industrial applications of acetic fermentation and acetic acid across different sectors is provided in Table 2.

Acetic fermentation also underpins the production of low-alcohol fermented beverages such as kombucha, water kefir, and kvass. In these systems, AAB form symbiotic consortia with yeasts (SCOBY), producing not only acetic and gluconic acids but also bacterial cellulose (e.g., *Komagataeibacter xylinus*). These metabolites contribute to acidity, carbonation, antioxidant capacity, and microbiota-modulating effects, enhancing the functional value of these beverages [9,54].

In the food industry, acetic fermentation is further exploited for the biosynthesis of natural flavors and esters. Acetic acid, acetaldehyde, and ethyl esters derived from fermentation are widely used in seasoning, dairy, and bakery applications as natural aromatic components. Acidification through acetic acid production also sup-ports biopreservation by inhibiting spoilage and pathogenic microorganisms, extending the shelf life of pickles, sauces, dressings, and marinades [43,61,81,82,96]. In this context, acetic fermentation provides a natural alternative to synthetic preservatives, aligning with consumer demand for clean-label and minimally processed foods.

At the industrial scale, acetic acid represents a high-volume platform chemical with global production exceeding 15 million tons annually, primarily for the synthesis of vinyl acetate monomer (VAM), purified terephthalic acid (PTA), and acetic anhydride [86,97]. While bulk acetic acid is still predominantly produced via petrochemical methanol carbonylation, food-grade and specialty acetic acid is exclusively obtained through microbial fermentation, and bio-based acetic acid is increasingly explored as a renewable alternative for polymer, textile, and pharmaceutical applications [86,98]. In the chemical industry, acetic acid serves as a precursor for the synthesis of polyethylene terephthalate (PET), cellulose acetate, and polyvinyl acetate, which are used in beverage packaging, photographic materials, coatings, and adhesives [99,100,101]. 

In the textile industry, acetic acid is used in both fiber production and fabric processing. Owing to its chemical properties, it plays a key role in dyeing processes, acting as a mordant that enhances color fastness and resistance to fading. It also functions as a pH regulator, adjusting the dye bath environment to meet the specific requirements of dyes and fiber types. Additionally, it is used in fabric cleaning, degreasing, and textile printing processes, where it facilitates dye binding and enhances pattern intensity and durability [24,34,87,90,102].

In cosmetics, acetic acid and its derivatives (esters and acetate salts) are incorporated into shampoos, conditioners, perfumes, and skincare formulations as pH adjusters, antimicrobial agents, and fragrance components [1,72,90,97]. In the pharmaceutical industry, acetic acid is used as a solvent, antiseptic, and synthetic intermediate in esterification and acetylation reactions, enabling the production of compounds with therapeutic properties. Due to its antimicrobial activity, it is also applied in wound cleansing and treatment of superficial infections [1,90].

**Table 2:** Industrial applications of acetic fermentation and acetic acid in the food, chemical, textile, cosmetic and pharmaceutical sectors.

Application Area	Example Products/Applications	Main Microorganisms	Fermentation Products/Effects	Technological Significance/Outcomes	Representative References
Vinegar production	Spirit vinegar, wine vinegar, apple cider vinegar, rice vinegar	*Acetobacter aceti*, *Komagataeibacter xylinus*, *Gluconacetobacter europaeus*	Acetic acid, water	Conversion of ethanol to acetic acid; pH reduction; flavor and aroma development; natural food preservation	[36,61,81,84,85,103,104]
Low-alcohol fermented beverages	Kombucha, water kefir, kvass	Symbiotic consortia of *Acetobacter*, *Komagataeibacter*, *Saccharomyces*, *Zygosaccharomyces*	Acetic acid, gluconic acid, bacterial cellulose	Functional beverages with antioxidant, detoxifying, and probiotic properties; SCOBY formation	[9,12,58,59,60,61,62,63,65,66]
Flavor and ester biosynthesis	Natural flavor concentrates, fruit and dairy flavoring compounds	*Acetobacter*, *Gluconobacter*, *Saccharomyces* spp.	Acetic acid, acetaldehyde, ethyl acetate, ethyl lactate	Generation of natural aromatic compounds for the dairy, seasoning, and bakery industries	[8,36,40,43,70,72,105]
Food preservation and bioprotection	Pickles, sauces, salad dressings, marinades	*Acetobacter* spp., *Lactobacillus* spp.	Acetic acid (acidification)	Growth inhibition of spoilage and pathogenic microorganisms; natural biopreservation; shelf-life extension	[1,4,6,36,65,106,107,108,109,110]
Chemical industry	Synthesis of polyethylene terephthalate (PET), cellulose acetate, polyvinyl acetate	Industrial oxidation systems using *Acetobacter* spp. or catalytic pathways	Acetic acid	Precursor for plastics, adhesives, and synthetic fibers; solvent in esterification and acetylation processes	[14,47,75,111,112]
Textile industry	Fiber dyeing and finishing, textile printing, degreasing	Industrial-grade acetic acid (chemical product)	Acetic acid	pH regulation in dye baths; mordant improving color fixation and fastness; cleaning and degreasing agent	[7,61,113]
Cosmetic industry	Hair care products, perfumes, skincare formulations	(industrial acetic acid and its esters)	Acetic acid, acetate esters, salts	Ingredient in shampoos and conditioners; pH adjuster; perfume component; antimicrobial additive	[47,61,95,104,110]
Pharmaceutical industry	Antiseptics, drug synthesis intermediates, solvents	(industrial acetic acid)	Acetic acid, acetyl derivatives	Reactant in acetylation and esterification; disinfectant and antimicrobial agent in medicinal preparations	[7,47,74,95,103,112]
Household and cleaning products	Window cleaners, dishwashing liquids, descalers	—	Diluted acetic acid	Removal of limescale, grease, and mineral deposits; eco-friendly cleaning and descaling agent	[61,75,95,103]

In household and institutional applications, diluted acetic acid is widely used as an eco-friendly cleaning agent. It is a common component of window cleaners, dishwashing liquids, and descaling formulations, where it effectively removes grease, mineral deposits, limescale, and rust [61,90,98].

Taken together, these examples demonstrate that acetic fermentation is not limited to vinegar production but constitutes a versatile biotechnological platform linking food processing with chemical manufacturing, materials science, pharmaceuticals, and sustainable consumer products.

## 4. Butyric Fermentation

In contrast to acetic fermentation, which relies on aerobic ethanol oxidation, butyric fermentation proceeds strictly under anaerobic conditions and redirects carbohydrate-derived carbon toward reduced short-chain fatty acids.

### 4.1. Biochemical Pathway and Key Microorganisms

Butyric fermentation is an anaerobic pathway of carbohydrate degradation in which the main end product is butyric acid (butanoic acid, CH_3_CH_2_CH_2_COOH), a short-chain volatile fatty acid. This process is carried out primarily by bacteria of the genera *Clostridium*, *Butyrivibrio* and *Butyribacterium*. Butyrate production has also been described in *Coprococcus*, *Eubacterium*, *Fusobacterium*, *Megasphaera*, *Roseburia* and *Sarcina*. Among these, *Clostridium* species are of particular importance due to their high fermentative potential and capacity to synthesize butyric acid from diverse substrates, including glucose, xylose, lactose and glycerol [114,115,116].

Metabolically, butyric fermentation begins with glycolysis via the EMP pathway, yielding pyruvate. Under strict anaerobic conditions, pyruvate is oxidatively decarboxylated to acetyl-CoA by pyruvate:ferredoxin oxidoreductase (PFOR), which functionally replaces the pyruvate dehydrogenase complex. This conversion is accompanied by the release of CO_2_ and the generation of reduced ferredoxin, with excess reducing equivalents dissipated as molecular hydrogen (H_2_). Acetyl-CoA serves as a central branching point. A fraction is converted to acetic acid via phosphotransacetylase (PTA) and acetate kinase (AK), generating ATP through substrate-level phosphorylation. The remaining acetyl-CoA condenses to acetoacetyl-CoA and is subsequently reduced to butyryl-CoA via β-hydroxybutyryl-CoA and crotonyl-CoA. Key enzymes include thiolase, β-hydroxybutyryl-CoA dehydrogenase, crotonase and butyryl-CoA dehydrogenase (linked to an electron-transferring flavoprotein, ETF) [114,115,116,117].

**Figure 3:**
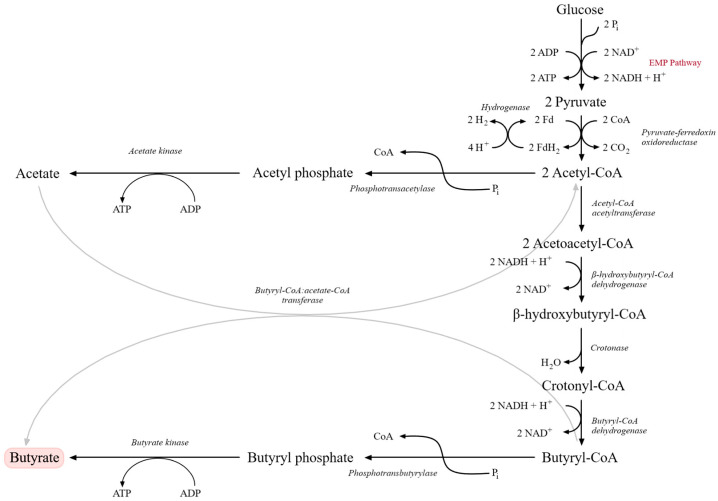
Metabolic pathway of butyric acid fermentation originating from glucose catabolism via the Embden–Meyerhof–Parnas (EMP) pathway. Glucose is converted to pyruvate, which is subsequently transformed into acetyl-CoA and further metabolized through acetoacetyl-CoA, β-hydroxybutyryl-CoA, crotonyl-CoA, and butyryl-CoA intermediates. Final conversion into butyrate occurs via butyryl phosphate. Parallel reactions involving acetate formation and ATP generation through substrate-level phosphorylation are also shown. Key enzymatic steps and electron transfer reactions involved in NADH oxidation and hydrogen production are indicated (adapted from [120]).

In the terminal step, butyryl-CoA is converted to butyric acid either via phosphotransbutyrylase (PTB) and butyrate kinase (BUK), through a butyryl phosphate intermediate, or via a CoA-transferase (CTF) that transfers CoA to acetate, producing butyrate and regenerating acetyl-CoA (Figure 3). Only some *Clostridium* strains possess both routes, and their relative contributions to total butyrate formation remain incompletely resolved [115,116,118]. Up to three moles of ATP per mole of glucose can be generated in butyric fermentation, which is relatively high for an anaerobic process. Most *Clostridium* species are heterofermentative, producing butyrate, acetate and, in smaller quantities, ethanol or isopropanol [117,118]. These metabolic characteristics explain both the technological potential and the process-related challenges associated with butyric fermentation in industrial systems.

Recent advances in metabolic engineering and synthetic biology have substantially expanded the biotechnological potential of butyric fermentation, particularly in solventogenic and butyrate-producing *Clostridium* species. Engineering efforts have focused on redirecting carbon flux toward butyrate and butanol by modulating key nodes such as the acetyl-CoA branch point (acetate vs. butyrate formation), strengthening reducing equivalent availability (ferredoxin/NADH balance), and reducing competing pathways that lead to lactate, acetone, or excessive acetate formation [115,116,119,120]. At the same time, CRISPR/Cas-based genome editing, together with systems-level analyses (transcriptomics, proteomics, and genome-scale metabolic models), has enabled targeted modification of acidogenesis/solventogenesis regulation, improved tolerance to butyric acid and butanol, and enhanced utilization of diverse substrates, including lignocellulosic sugars and glycerol [121,122]. Adaptive laboratory evolution and dynamic pathway control are increasingly combined with in situ product removal strategies to mitigate end-product inhibition and improve process productivity, supporting the transition of butyric fermentation from a classical anaerobic pathway to a rationally optimized platform for the sustainable production of short-chain fatty acids and bio-based solvents [123,124,125].

### 4.2. Industrial Applications

In industrial practice, butyric fermentation has been exploited across the food, chemical, energy, and pharmaceutical sectors due to its capacity to generate butyrate, solvents, and other value-added metabolites. Although pure butyric acid has a pungent odor, its esters exhibit pleasant fruity aromas and are widely used as flavoring agents in food, confectionery and perfumery. In dairy technology, uncontrolled butyric fermentation by *Clostridium tyrobutyricum* can cause late blowing in cheese, leading to quality defects and economic losses. Conversely, controlled butyrate formation contributes to characteristic flavor profiles in some traditional cheeses [126,127,128]. Representative industrial applications of butyric fermentation and butyric acid are summarized in Table 3.

In the chemical and materials industries, butyric acid and its derivatives are important intermediates in the synthesis of plastics and textile fibers, improving resistance to heat, light and mechanical stress. Cellulose acetate butyrate (CAB), a thermoplastic material derived from butyric acid, combines flexibility, UV stability and solubility in organic solvents, making it suitable for durable coatings, films and specialty polymers [47,114,116].

Butyric fermentation also underlies the acetone—butanol—ethanol (ABE) process, in which *Clostridium acetobutylicum* and *C. beijerinckii* produce solvents such as butanol, acetone and ethanol. Butanol is regarded as a promising advanced biofuel due to its high energy density and favorable combustion properties, emphasizing the importance of butyric fermentation in renewable energy systems [7,34,116].

Butyrate and its salts (e.g., sodium and calcium butyrate) are widely used in pharmaceuticals, animal nutrition, cosmetics and the synthesis of bioplastics and bioesters. In animal feed, butyrate-based additives serve as alternatives to antibiotic growth promoters, supporting gut health, nutrient absorption and immune function [75,114,115,129].

In environmental biotechnology, butyrate-producing bacteria participate in the degradation of biomass and organic waste, contributing to biogas generation and the production of organic acids that can be upgraded to biofuels or biochemical intermediates [75,123,130]. *Clostridium butyricum* is also considered a probiotic capable of producing butyrate in the colon, where it supports epithelial cell metabolism, exerts anti-inflammatory and anticarcinogenic effects, and promotes a beneficial microbiota composition [11,129,131].

In medicine, butyric acid has therapeutic potential in gastrointestinal diseases, colorectal cancer and hemoglobinopathies [132,133,134]. It induces differentiation and reduces proliferation of cancer cells, promotes regulatory T cell (Treg) generation and modulates immune responses [133,135,136]. By enhancing mitochondrial function, oxidative phosphorylation and fatty acid β-oxidation, butyrate also shows neuroprotective properties [137,138]. Several butyrate derivatives with antithyroid, anesthetic and vasoconstrictive activities have been developed, and new acyloxyalkyl butyrate prodrugs are under clinical investigation [139,140].

**Table 3:** Representative applications of butyric fermentation and butyric acid in food, energy, chemical and pharmaceutical industries.

Application Area	Example Products/Applications	Main Microorganisms	Fermentation Products/Effects	Technological Significance/Outcomes	Representative References
Food and flavor industry	Butter flavoring, cheese production, aroma compounds, fruit esters	*Clostridium butyricum*, *Clostridium tyrobutyricum*, *Butyrivibrio fibrisolvens*	Butyric acid, ethyl butyrate, butyl butyrate	Natural flavor generation (buttery, fruity notes); improvement of aroma profiles; undesirable in cheese spoilage (“late blowing”)	[43,114,115,117,128,141,142,143,144,145]
Chemical and materials industry	Cellulose acetate butyrate (CAB), plasticizers, textile fibers	*Clostridium acetobutylicum*, *Clostridium beijerinckii*	Butyric acid, butanol, acetone, esters	Synthesis of thermoplastics, coatings, and resins; improved material flexibility, UV stability, and solvent resistance	[25,33,47,74,75,111,114,143]
Biofuel and solvent production (ABE process)	Butanol, acetone, ethanol	*Clostridium acetobutylicum*, *C. beijerinckii*, *C. pasteurianum*	Butanol, acetone, ethanol	Production of renewable solvents and biofuels; butanol as a high-energy, low-volatility gasoline substitute	[14,33,47,75,111,112,114,143]
Animal nutrition and feed additives	Livestock feed, poultry supplements	*Clostridium butyricum*, *Butyrivibrio fibrisolvens*	Sodium butyrate, calcium butyrate	Replacement for antibiotic growth promoters; enhancement of gut health, nutrient absorption, and immunity	[45,100,112,115,128,134,146,147]
Pharmaceutical and medical applications	Therapeutics for gut disorders, cancer, hemoglobinopathies	*Clostridium butyricum*	Butyric acid, butyrate derivatives	Anti-inflammatory, anticarcinogenic, and neuroprotective effects; induction of cell differentiation; modulation of immune response	[43,100,115,128,134,146,148,149,150]
Probiotic and microbiome modulation	Probiotic supplements, intestinal health products	*Clostridium butyricum*, *Butyrivibrio fibrisolvens*	Butyric acid (SCFA)	Regulation of gut microbiota; stimulation of epithelial regeneration; trophic effect on colonocytes	[45,100,110,112,115,134,146,149,151,152]
Environmental biotechnology	Biogas and biohydrogen production, waste valorization	*Clostridium butyricum*, *C. pasteurianum*	H_2_, CO_2_, volatile fatty acids	Conversion of organic waste into biogas and organic acids; sustainable bioenergy recovery and waste reduction	[33,50,75,109,111,114,123,130,143,153]
Cosmetic industry	Skin and hair care formulations	Industrially derived butyric esters	Butyric esters, butyrate salts	Use in fragrance formulations; moisturizing and conditioning properties; pH regulation	[95,100,110,115,134,146]

## 5. Lactic Fermentation

Unlike butyric fermentation, which primarily directs carbon toward reduced short-chain fatty acids and solvents, lactic fermentation channels carbohydrate metabolism toward lactate formation and rapid acidification of the environment.

### 5.1. Metabolic Pathways and Key Microbial Groups

**Figure 4:**
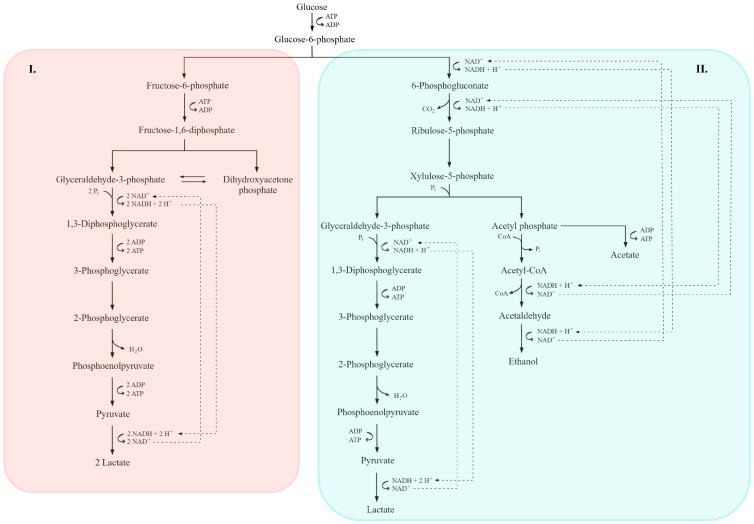
Comparative overview of homofermentative (**I**) and heterofermentative (**II**) lactic acid fermentation pathways. In the homofermentative route (**I**), glucose is metabolized via the Embden–Meyerhof–Parnas (EMP) pathway, yielding pyruvate and subsequently lactate as the main end product, along with ATP generation. In the heterofermentative route (**II**), glucose is processed through the 6-phosphogluconate/phosphoketolase pathway, leading to the formation of lactate, ethanol or acetate, and CO_2_. Key intermediates, redox reactions, and ATP-yielding steps are indicated. Dashed lines denote NADH/NAD^+^ electron transfer pathways, while the shaded areas highlight the distinct metabolic branches of each fermentation type (adapted from [7,36,156]).

Lactic acid fermentation is carried out by both homo- and heterofermentative lactic acid bacteria (LAB) [4,54,154]. Homolactic LAB metabolize hexoses (primarily glucose) via the EMP pathway (Figure 4), yielding two molecules of lactate per molecule of glucose [7,36,155,156]. Glucose is converted to pyruvate, which is reduced to lactate by lactate dehydrogenase with concomitant re-oxidation of NADH to NAD^+^, resulting in a net gain of two ATP per mole of glucose [157]. Strict homolactic fermentation of glucose does not generate CO_2_ [158]. Representative homolactic LAB include *Lactiplantibacillus plantarum* (formerly *Lactobacillus plantarum*), *Pediococcus pentosaceus*, *Lactococcus lactis* and *Streptococcus thermophilus* [112,142,156,159].

Homolactic pathway:

C_6_H_12_O_6_ (glucose) → 2 CH_3_-CHOH-COOH (lactic acid)

In contrast, heterofermentative LAB can ferment both hexoses and pentoses. For hexoses, they employ the phosphoketolase branch of the pentose phosphate pathway (PPP; Figure 3), which requires ribulose-5-phosphate 3-epimerase and phosphoketolase. This route typically yields equimolar lactate, ethanol (or acetate) and CO_2_ [106,157,158,160,161]. Obligate heterofermenters lack key EMP enzymes—most notably fructose-bisphosphate aldolase and triose phosphate isomerase—thus channeling carbon exclusively through the phosphoketolase pathway. Typical heterofermentative genera include *Leuconostoc*, *Oenococcus*, *Levilactobacillus* (e.g., *L. brevis*) and *Limosilactobacillus* (e.g., *L. fermentum*) [142,158,159,161].

Heterofermentative phosphoketolase pathway:

C_6_H_12_O_6_ (glucose) → CH_3_-CHOH-COOH (lactic acid) + C_2_H_5_OH (ethanol) + CO_2_ [106,157,161].

(In the presence of external electron acceptors, ethanol can be replaced by acetate with additional ATP formation) [158,161]. The balance between homo- and heterofermentative metabolism, ATP yield, and by-product formation directly influences both the technological performance of lactic acid bacteria and the characteristics of fermented products [106,142,160,161].

In recent years, lactic acid bacteria have become prominent targets of metabolic engineering and synthetic biology aimed at improving lactic acid yield, optical purity, robustness, and industrial scalability [155,156,158]. Engineering strategies have focused on controlling the stereospecificity of lactate dehydrogenases to selectively produce optically pure L- or D-lactic acid, which is critical for high-performance polylactic acid (PLA) synthesis [162]. Additional approaches include redirection of carbon flux toward lactate by minimizing by-product formation (e.g., ethanol, acetate, or CO_2_), enhancement of acid tolerance through membrane and stress-response engineering, and optimization of redox balance [112,157,159].

Advances in genome sequencing, CRISPR/Cas-based genome editing, and systems-level analyses have enabled precise modification of industrial LAB strains such as *Lactiplantibacillus plantarum*, *Lactococcus lactis*, and *Streptococcus thermophilus*. These tools have facilitated the development of strains with improved substrate utilization, resistance to bacteriophages, and stable performance under low-pH and high-product conditions [27,158,163]. Together with adaptive laboratory evolution and process-integrated control strategies, these innovations support the transition of lactic fermentation from a traditional food process to a rationally engineered platform for sustainable production of organic acids, biopolymers, and functional food ingredients [123,130].

### 5.2. Industrial and Health-Related Applications

From an industrial and technological perspective, lactic fermentation has been widely applied in food processing, health-oriented products, and industrial biotechnology due to its efficiency, safety, and functional metabolite profile. LAB convert carbohydrates, mainly glucose and lactose, into lactic acid and secondary metabolites such as diacetyl, acetaldehyde, hydrogen peroxide and bacteriocins [7,36,157,158]. These compounds contribute to the sensory profile, stability and microbiological safety of fermented foods by lowering pH and inhibiting pathogenic and spoilage microorganisms [110,142]. The major applications of lactic fermentation and lactic acid across food, medical, and materials-related sectors are summarized in Table 4.

Lactic fermentation underlies the production of numerous fermented foods, including dairy products (yogurt, kefir, buttermilk, cheese), vegetables (sauerkraut, kimchi, pickled cucumbers), meats (dry-cured sausages, salami), cereal-based products (sourdough) and plant-based beverages (soy, oat and beetroot drinks) [9,79]. LAB such as *Lactobacillus delbrueckii* subsp. *bulgaricus*, *S. thermophilus*, *L. plantarum* and *Leuconostoc mesenteroides* are essential for improving quality, nutritional value and sensory characteristics [129,142,159].

Beyond traditional fermentation, LAB are central to the development of functional and probiotic foods. Strains such as *Lactobacillus rhamnosus* GG, *L. casei* and *Bifidobacterium bifidum* are associated with health benefits, including support of the intestinal microbiota, enhanced mineral absorption, synthesis of B-group vitamins and modulation of immune responses [110,129,146,149,154,164,165]. In the food industry, lactic acid acts as a natural preservative, acidulant, pH regulator, flavor enhancer, cryoprotectant and prebiotic component [106,166]. Owing to its antibacterial and antioxidant properties, it is also used as a disinfectant and stabilizer in fermented food production [71,102,110,155,158].

In the chemical industry, lactic acid is a renewable platform chemical for the production of biopolymers, particularly polylactic acid (PLA), which is an environmentally friendly alternative to petroleum-based plastics. PLA is used in biodegradable films, fibers and packaging materials, contributing to green chemistry and circular economy strategies [7,14,47,50]. Lactic acid is also used in the synthesis of lactate esters, acrylic acid, propylene oxide, acetaldehyde and propylene glycol, which are widely applied in cosmetic, pharmaceutical and chemical products [47,155].

In dermatology and cosmetology, lactic acid is valued for its moisturizing, exfoliating and brightening properties. It is incorporated into anti-acne, anti-ageing and hydrating formulations as well as intimate hygiene products. Acting as a humectant, it binds water in the stratum corneum and improves skin elasticity and appearance [49,155,166].

In medicine and pharmacy, lactic acid and its salts (lactates) are widely used due to their biocompatibility, low toxicity and involvement in natural metabolic pathways [7,155]. They form key components of infusion solutions such as Ringer’s lactate, which help maintain acid–base balance and restore electrolytes in patients with dehydration or metabolic acidosis [7]. Calcium, magnesium and zinc lactates are used in dietary supplements and pharmaceutical formulations due to their high bioavailability and stabilizing properties [110,146,165]. 

A major pharmaceutical application of lactic acid is its role as a monomer for biodegradable polymers, including PLA and its copolymers such as poly(lactide-co-glycolide) (PLGA). These materials are used in biodegradable implants, surgical sutures and controlled drug delivery systems, enabling gradual and targeted release of active compounds, particularly in oncology, endocrinology and immunotherapy [7,14,36,47]. Lactic acid-derived materials are also used to fabricate biodegradable scaffolds for tissue engineering, supporting bone, muscle and skin regeneration [7,47,167]. Their biodegradability and renewable origin align with sustainable development principles and offer an eco-friendly alternative to conventional synthetic polymers [9,14,50,99,101].

**Table 4:** Lactic fermentation and lactic acid: major applications in food, cosmetics, pharmaceuticals and materials science.

Application Area	Function/Role of Lactic Fermentation or Lactic Acid	Key Microorganisms/ Compounds	Industrial or Health Relevance	Selected References
Food Fermentation and Preservation	Conversion of carbohydrates (mainly glucose and lactose) into lactic acid, diacetyl, acetaldehyde, hydrogen peroxide, and bacteriocins; acidification of the environment inhibits spoilage and pathogenic microorganisms.	*Lactobacillus delbrueckii* subsp. *bulgaricus*, *Streptococcus thermophilus*, *Lactiplantibacillus plantarum*, *Leuconostoc mesenteroides*	Production of fermented foods (yogurt, kefir, cheese, sauerkraut, kimchi, sourdough); enhanced safety, shelf-life, and sensory quality.	[5,13,27,36,71,77,79,106,155,156,159,168,169,170,171,172,173,174]
Functional and Probiotic Foods	Support of intestinal microbiota; synthesis of B-group vitamins; enhancement of mineral absorption; immune modulation.	*Lactobacillus rhamnosus* GG, *L. casei*, *Bifidobacterium bifidum*	Development of health-promoting foods with probiotic activity; improvement of gastrointestinal and immune health.	[6,11,96,110,129,131,143,146,147,149,151,152,154,165,175,176,177,178,179,180,181,182,183,184,185]
Food Industry (Technological Additive)	Acts as a natural preservative, acidulant, pH regulator, flavor enhancer, cryoprotectant, and prebiotic component; provides antimicrobial and antioxidant protection.	Lactic acid; bacteriocins (e.g., nisin); hydrogen peroxide	Improves food quality, safety, and texture; stabilizes emulsions; inhibits spoilage flora.	[4,17,22,28,36,71,72,106,127,155,157,158,159,161,186,187,188]
Biopolymer Production	Precursor for polylactic acid (PLA) synthesis, an eco-friendly biodegradable polymer replacing petrochemical plastics.	Lactic acid (from *Lactobacillus* fermentation)	PLA used in films, fibers, and packaging materials; supports circular economy and green chemistry.	[7,14,25,33,43,47,74,75,124,161]
Chemical Industry	Intermediate for synthesis of lactate esters, acrylic acid, propylene oxide, acetaldehyde, and propylene glycol.	Lactic acid and its derivatives	Production of solvents, adhesives, surfactants, and coatings for cosmetics, pharmaceuticals, and polymers.	[7,14,47,74,75,155,161,166]
Cosmetics and Dermatology	Humectant, exfoliant, and brightening agent in skincare; promotes hydration, elasticity, and renewal of the stratum corneum.	Lactic acid and its salts	Used in anti-aging, anti-acne, and moisturizing formulations; improves skin tone and appearance.	[107,110,146,148,155]
Medicine and Pharmacy	Ingredient in infusion solutions (e.g., Ringer’s lactate); component of mineral supplements; stabilizer in drug formulations.	Calcium, magnesium, and zinc lactates	Rehydrates and maintains acid–base balance; enhances bioavailability of minerals.	[7,47,148,155]
Pharmaceutical Biotechnology	Building block for biodegradable polymers such as PLA and PLGA used in drug delivery systems, sutures, and implants.	Lactic acid monomers and copolymers	Enables controlled drug release, tissue compatibility, and gradual biodegradation in vivo.	[7,33,43,47,112,161]
Tissue Engineering and Regenerative Medicine	Source material for biodegradable scaffolds supporting bone, muscle, and skin regeneration.	PLA and its copolymers; lactic acid-based composites	Sustainable alternative to petrochemical polymers; biocompatible matrices for tissue growth.	[7,9,14,33,43,47,74,161]

## 6. Propionic Fermentation

In contrast to lactic fermentation, propionic fermentation represents a metabolically linked process in which lactate and other reduced substrates are further converted into propionic acid, acetate, and CO_2_ under anaerobic conditions. 

### 6.1. Wood–Werkman (Dicarboxylic Acid) Pathway and Key Microorganisms

Propionic acid fermentation is an anaerobic metabolic process in which substrates such as glucose, glycerol or lactic acid are converted into propionic acid, accompanied by acetic acid and CO_2_ formation [111,119]. This process is characteristic of several groups of anaerobic bacteria, among which *Propionibacterium* species have the highest industrial relevance. These microorganisms exhibit high fermentative efficiency and a broad substrate spectrum (Table 5) [189]. Other bacteria capable of propionic fermentation include *Clostridium propionicum* and selected species of *Veillonella*, *Selenomonas*, *Megasphaera*, *Fusobacterium* and *Bacteroides*, which typically show lower yields and a narrower substrate range [111,119]. 

Stoichiometrically, propionic acid fermentation can be expressed as follows:

For glucose,

C_6_H_12_O_6_ → 4/3 CH_3_CH_2_COOH + 2/3 CH_3_COOH + 2/3 CO_2_ + 4/3 H_2_O + 4 ATP

For lactic acid,

CH_3_CHOHCOOH → 2/3 CH_3_CH_2_COOH + 1/3 CH_3_COOH + 1/3 CO_2_ + 2/3 H_2_O + ATP

Propionic acid can be produced via three main metabolic routes: (1) the Wood–(dicarboxylic acid) cycle, (2) the acrylate pathway and (3) the 1,2-propanediol pathway [189]. In *Propionibacterium* spp., the predominant route is the Wood– pathway. It starts from pyruvate, generated via glycolysis, which can follow two alternative routes:

(i) Carboxylation to oxaloacetate: this initiates the Wood–Werkman cycle and leads to propionic acid. (ii) Decarboxylation to acetyl-CoA: this serves as the precursor for acetic acid.

In the first route, pyruvate is converted to oxaloacetate in a biotin-dependent carboxyl transfer reaction catalyzed by methylmalonyl-CoA carboxytransferase, which transfers a carboxyl group from methylmalonyl-CoA to pyruvate, yielding oxaloacetate and propionyl-CoA. Oxaloacetate is then reduced to malate, dehydrated to fumarate and reduced to succinate. Succinate is converted to succinyl-CoA by succinyl-CoA synthetase and rearranged via a vitamin B_12_-dependent methylmalonyl-CoA mutase to methylmalonyl-CoA and back to propionyl-CoA. Finally, propionyl-CoA is converted to propionic acid with the release of CoA by a CoA-transferase [74,88,98,118,189,201].

Parallel to this, a portion of pyruvate may be converted into acetyl-CoA, followed by the formation of acetic acid. This occurs through pyruvate dehydrogenase complex–catalyzed decarboxylation of pyruvate to acetyl-CoA, which is subsequently converted into acetate via phosphotransacetylase (PTA) and acetate kinase (AK) reactions, yielding ATP as a byproduct. Consequently, acetic acid is produced as a secondary metabolite of propionic acid fermentation [88,98,118,189,201]. 

**Figure 5:**
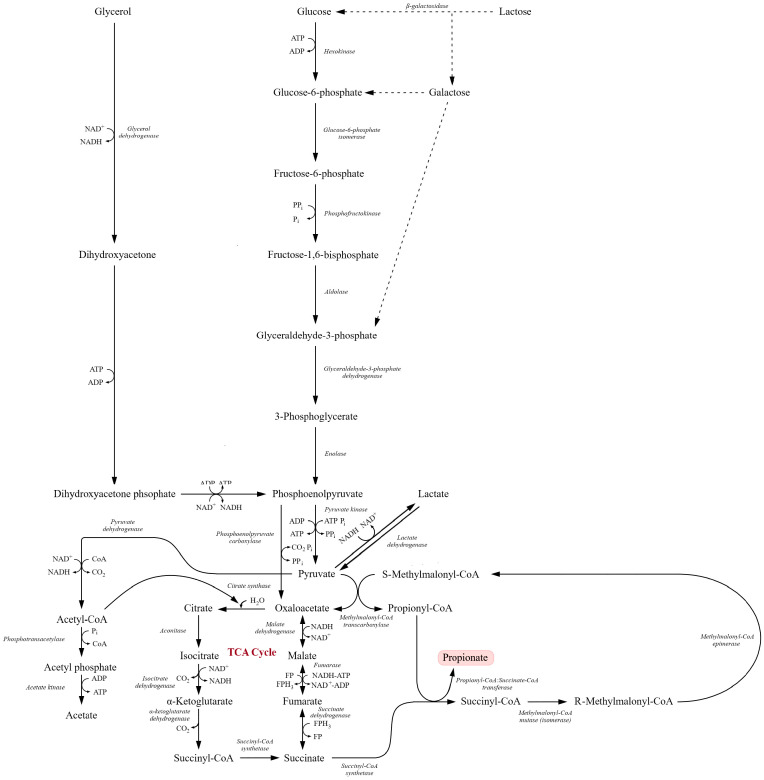
Integrated metabolic pathways involved in the conversion of carbohydrates and glycerol into propionate via the succinate–propionate pathway. Glucose, galactose, and glycerol are converted to dihydroxyacetone phosphate and subsequently processed through glycolysis to phosphoenolpyruvate and pyruvate. Pyruvate is then metabolized through the tricarboxylic acid (TCA) cycle to oxaloacetate, malate, fumarate, succinate, and succinyl-CoA. Further reactions lead to the formation of methylmalonyl-CoA intermediates and ultimately propionyl-CoA and propionate. Key enzymatic steps, redox reactions, ATP-generating conversions, and metabolic branch points linking glycolysis, glycerol utilization, and the TCA cycle are indicated (adapted from [88,189,201]).

A schematic representation of the propionic acid fermentation pathway and the key enzymes involved is shown in Figure 5 [74,88,98,118,189]. While the Wood–Werkman pathway predominates in *Propionibacterium* species, alternative metabolic routes have evolved in other anaerobic bacteria to support propionate formation under different physiological and ecological conditions.

In recent years, propionibacteria have increasingly been explored as targets for metabolic engineering and systems-guided strain improvement to enhance propionic acid productivity, reduce by-product formation (especially acetate), and improve tolerance to product inhibition [111,119,189,201]. A major limitation of industrial propionate production is the strong inhibitory effect of propionic acid on cell growth and redox homeostasis; therefore, contemporary strategies focus on improving acid tolerance and export capacity, as well as optimizing intracellular redox balance and ATP conservation in the Wood–Werkman cycle [89,121,193]. In parallel, pathway-level interventions aimed at shifting carbon flux toward propionyl-CoA (and away from acetyl-CoA/acetate) have been investigated, including regulation of key nodes such as pyruvate utilization, succinate conversion steps, and CoA-transferase reactions [192,194,202].

Advances in genome sequencing, metabolic modeling, and multi-omics analyses have improved understanding of propionibacterial physiology and facilitated rational process optimization, particularly for *Propionibacterium freudenreichii* and *P. acidipropionici*, which are also major industrial producers of vitamin B_12_ [189,191,203]. Emerging synthetic biology approaches—including targeted genome editing, adaptive laboratory evolution, and engineered co-cultures that couple lactate-producing LAB with propionate producers—are increasingly used to improve stability, yields, and substrate flexibility, supporting the development of propionic fermentation as a sustainable platform for food preservation and bio-based chemicals [28,123,204].

### 6.2. Alternative Propionate Pathways (Acrylate and 1,2-Propanediol)

**Figure 6:**
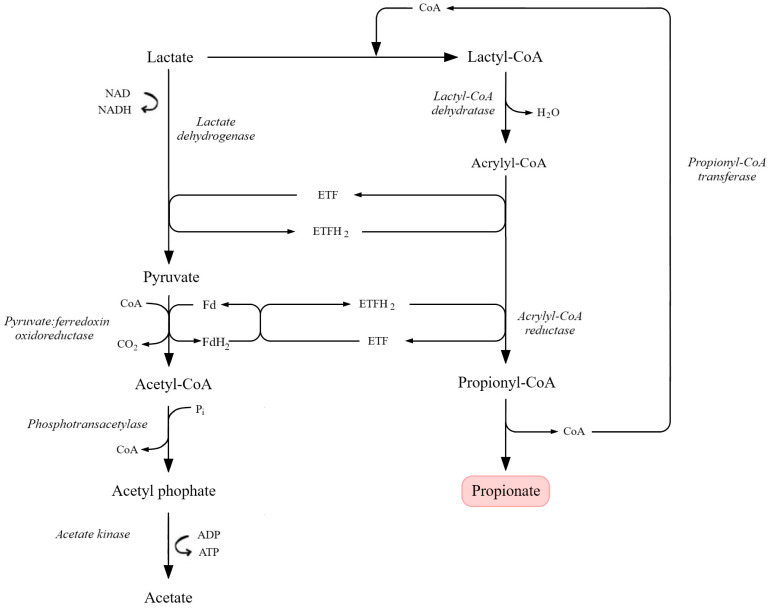
Lactate-based acrylate pathway leading to propionate formation. Lactate is oxidized to pyruvate via lactate dehydrogenase, with subsequent conversion to acetyl-CoA by pyruvate:ferredoxin oxidoreductase. In the acrylate branch, lactyl-CoA is dehydrated to acrylyl-CoA, which is further reduced to propionyl-CoA by acrylyl-CoA reductase in an electron-transferring flavoprotein (ETF/ETFH_2_)-dependent system. Propionyl-CoA is then converted to propionate through CoA transferase activity. Parallel conversion of acetyl-CoA to acetate via acetyl phosphate also contributes to ATP generation. Key enzymatic reactions, redox cycles, and metabolite flow are indicated (adapted from [201]).

The acrylate pathway is an alternative propionic acid fermentation route used by some obligate anaerobes, notably *C. propionicum*. It begins with the conversion of pyruvate to lactate via L-lactate dehydrogenase. Propionyl-CoA transferase then transfers CoA from propionyl-CoA to lactate, producing lactoyl-CoA and releasing propionic acid, the main product of this pathway. Lactoyl-CoA is dehydrated by lactoyl-CoA dehydratase to acryloyl-CoA, a toxic intermediate. To minimize toxicity, acryloyl-CoA is rapidly reduced back to propionyl-CoA by acryloyl-CoA reductase. The pathway thus functions as a cyclic process with internal CoA recycling, in which propionyl-CoA transferase plays a central dual role (Figure 6) [88,189,193,201].

Because acryloyl-CoA is cytotoxic, bacteria must maintain its intracellular concentration at very low levels. This becomes particularly challenging at elevated pH, when redox balance is disturbed. Under such conditions, microorganisms redirect carbon flux toward acetic acid formation at the expense of propionic acid, rendering the acrylate pathway less efficient. The metabolic burden associated with rapid detoxification of acryloyl intermediates further reduces overall fermentation yield [89,189,201,202,205].

The 1,2-propanediol fermentation pathway constitutes another route to propionic acid. Several bacteria, including *Salmonella enterica*, *Roseburia inulinivorans* and representatives of *Lactobacillus*, *Ruminococcus obeum* and *Bacteroides thetaiotaomicron*, can synthesize 1,2-propanediol from various carbon sources such as deoxy sugars (e.g., fucose and rhamnose), dihydroxyacetone or lactic acid [122,206,207].

**Figure 7:**
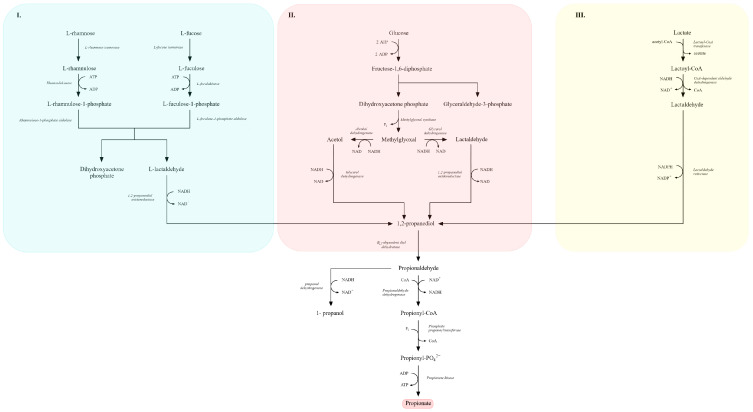
Metabolic pathways leading to the formation of 1,2-propanediol and propionate from sugars, sugar-derived deoxyhexoses, and lactate. Panel (**I**) shows the catabolism of L-rhamnose and L-fucose to dihydroxyacetone phosphate and L-lactaldehyde via L-rhamnulose-1-phosphate and L-fuculose-1-phosphate intermediates. Panel (**II**) presents the conversion of glucose-derived triose phosphates into acetol, methylglyoxal, and lactaldehyde, followed by their reduction to 1,2-propanediol. Panel (**III**) illustrates the lactate-dependent pathway in which lactoyl-CoA and lactaldehyde serve as precursors for 1,2-propanediol formation. The central pathway depicts the downstream conversion of 1,2-propanediol to propionaldehyde, propionyl-CoA, propionyl-phosphate, and finally propionate. Key enzymatic steps, redox reactions, and metabolic branch points between carbohydrate, deoxyhexose, and lactate catabolism are indicated (adapted from [189,201]).

Three main microbial routes for 1,2-propanediol biosynthesis are described (Figure 7):

I. Deoxyhexose pathway—L-rhamnose or L-fucose is metabolized to 1,2-propanediol. For example,

L-fucose → L-fuculose → fuculose-1-phosphate → L-lactaldehyde → 1,2-propanediol, via L-fucose isomerase, L-fuculokinase, L-fuculose-1-phosphate aldolase and lactaldehyde reductase [122,207].

II. Methylglyoxal pathway—dihydroxyacetone phosphate (DHAP) is converted to methylglyoxal and subsequently reduced to 1,2-propanediol. Due to the cytotoxicity of methylglyoxal, this route is less favored [122,208].

III. Lactic acid pathway—lactic acid is converted to lactoyl-CoA, reduced to lactaldehyde and then to 1,2-propanediol. This pathway, often active under acidic conditions, is considered most attractive for industrial applications because it does not generate toxic intermediates and does not require expensive substrates [200,204].

After synthesis, 1,2-propanediol can be further converted to propionic acid. It is dehydrated to propionaldehyde by propanediol dehydratase, then oxidized to propionyl-CoA and reduced to propanol by propionaldehyde and propanol dehydrogenases, respectively. Propionyl-CoA is finally converted to propionyl phosphate and then to propionic acid by phosphotransacylase and propionate kinase [189,192,200]. 

The diversity of propionate-forming pathways, their energetic efficiency, and their dependence on substrate availability collectively determine the industrial and technological relevance of propionic fermentation.

### 6.3. Industrial Applications

Building on the metabolic frameworks described above, propionic fermentation has found widespread application in food processing, biotechnology, and chemical industries due to the preservative, functional, and platform-chemical properties of propionic acid [7,54,203]. The best-known example is the ripening of Swiss-type cheeses (e.g., Emmental, Maasdam, Gruyère), in which *Propionibacterium freudenreichii* subsp. *shermanii* converts lactic acid into propionic and acetic acids and CO_2_. Gas evolution forms the characteristic eyes in the cheese, while organic acids contribute to the nutty aroma and mild flavor. Propionic acid also inhibits molds and spoilage bacteria, extending shelf life [36,144]. An overview of the major industrial applications of propionic fermentation and propionic acid is provided in Table 6.

In the wider food industry, propionic acid and its salts—sodium, calcium, potassium and ammonium propionates—are used as natural preservatives due to strong antibacterial and antifungal properties. They inhibit species such as *Aspergillus flavus*, *Bacillus* spp., *Salmonella* spp. and yeasts, and in combination with lactic and acetic acids effectively suppress *Listeria monocytogenes* and other foodborne pathogens [74,98,189]. Propionates are therefore used in bakery products, confectionery, cheeses and silage to improve microbial stability and safety.

Propionic acid (CH_3_CH_2_COOH) is a three-carbon carboxylic acid, miscible with water and many organic solvents, and has been granted GRAS status by the U.S. Food and Drug Administration. Beyond food, it is used in chemical, cosmetic, polymer and pharmaceutical industries [111,119]. In biotechnology, propionic fermentation serves as the basis for industrial vitamin B_12_ (cobalamin) production by *P. freudenreichii* and *P. acidipropionici*. Microbial cobalamin is widely used in pharmaceuticals and functional foods [79,189].

In the chemical industry, propionic acid acts as an important intermediate in the synthesis of various organic compounds, including herbicides and pesticides used in crop protection. Due to its chemical reactivity, it is also used as a precursor for the synthesis of propionate esters, which serve as components in protective coatings, industrial varnishes, and organic solvents. These esters are valued for their volatility, film-forming properties, and durability, making them crucial in the paint and coatings industry [74,98].

In the construction materials and cleaning products sector, propionic acid is applied for its antimicrobial activity. It is added to paints, adhesives, detergents, and impregnating agents to prevent the growth of bacteria, molds, and fungi on surfaces, thereby enhancing material durability and improving hygienic safety [119].

In the plastics industry, propionic acid and its esters are key intermediates for cellulose-based fibers and biodegradable polymers. Cellulose propionate combines favorable mechanical, optical and moisture-resistance properties, making it suitable for films, packaging, photographic layers and decorative components, and it is considered a next-generation biopolymer [100,111,209].

In the cosmetic industry, propionate salts are used as fragrance bases and natural preservatives in skincare formulations. When combined with butyl rubber, they improve the texture, elasticity, and stability of cosmetic emulsions, while their antimicrobial activity reduces the need for synthetic preservatives [119].

In pharmaceutical and veterinary applications, propionic acid and its salts—especially sodium propionate—are used as auxiliary and therapeutic substances. Due to their antibacterial and anti-inflammatory properties, they are applied in the treatment of skin infections, fungal diseases, and mucosal inflammations. Propionates are also components of ophthalmic preparations used to treat conjunctivitis and are included in antiseptic and anti-inflammatory drugs. In veterinary medicine, they are used in hygiene preparations for livestock, protecting against infections of the skin and hooves [74,98,119]. The multifunctionality of propionic acid and its derivatives makes them a crucial link between biotechnology, chemical, pharmaceutical, and material industries. Owing to their potential integration into sustainable bioproduction processes, propionic acid is considered a key biocomponent of the future, aligning with the principles of green chemistry and the circular economy [9,111].

## 7. Comparative Roles of Key Microorganisms Across Fermentation Systems

Having reviewed the biochemical pathways, microbial ecology, and industrial applications of alcoholic, acetic, butyric, lactic, and propionic fermentations, it becomes evident that although certain microorganisms occur across multiple fermentation systems, their metabolic roles and technological relevance differ substantially depending on ecological context and process conditions. *S. cerevisiae* exemplifies this functional plasticity, acting as the primary biocatalyst of alcoholic fermentation by converting sugars into ethanol and CO_2_ via fermentative glycolysis, while simultaneously generating a wide spectrum of aroma-active secondary metabolites that define beverage quality [8,20,26,57]. In contrast, in lactic and mixed-culture fermentations, yeasts such as *S. cerevisiae*, *Kluyveromyces* spp., and *Torulaspora* spp. typically play auxiliary roles, contributing to redox balance, oxygen scavenging, vitamin release, and the formation of volatile compounds that support lactic acid bacteria growth and enhance sensory complexity rather than serving as dominant fermentative agents [46,54,142,173].

Lactic acid bacteria represent the principal acidifying microorganisms in lactic fermentation, where they drive rapid pH reduction, microbial stabilization, and product safety through lactic acid and bacteriocin production [37,106,157,159]. However, in alcoholic and acetic fermentations, LAB generally constitute secondary microbiota, influencing microbial succession, flavor development, and maturation processes, particularly in wine, beer, and traditional fermented beverages [41,57,64,87]. A further context-dependent functional shift is observed for acetic acid bacteria, which dominate ethanol oxidation during acetic fermentation but appear as minor or late-stage contributors in alcoholic or mixed fermentations, where they modulate acidity and aroma rather than serving as primary producers [83,87,95].

In propionic fermentation, *Propionibacterium* species assume a specialized role by converting lactate into propionic and acetic acids and CO_2_ via the Wood–Werkman pathway, thereby linking lactic and propionic fermentations both metabolically and technologically, as exemplified by Swiss-type cheese ripening [126,189,193,195]. Collectively, these examples demonstrate that microbial functionality in fermentation cannot be interpreted solely at the species level but must be evaluated within a dynamic ecological framework shaped by substrate availability, interspecies interactions, and process design [27,54,173]. This integrative perspective provides a conceptual synthesis of microbial roles across fermentation systems and underpins the diverse applications discussed throughout this review.

## 8. Conclusions and Future Perspectives

Fermentation, one of humanity’s oldest biotechnological tools, remains central to both traditional food production and cutting-edge industrial biotechnology. This review has summarized the biochemical foundations, microbial ecology and industrial relevance of the five major fermentation types: alcoholic, acetic, butyric, lactic and propionic fermentation. Despite their diversity, these processes share common features: they enable energy conservation under anaerobic or microaerophilic conditions, generate organic acids and alcohols as key metabolites, and transform raw materials into products with enhanced shelf life, safety, sensory quality and functional value.

The diversity of microbial communities plays a decisive role in shaping the sensory properties, quality, and consistency of fermented products. In spontaneous and mixed-culture fermentations, complex consortia of yeasts, lactic acid bacteria, acetic acid bacteria, and other microorganisms engage in cross-feeding, metabolic cooperation, and competitive interactions that generate rich and distinctive flavor profiles. At the same time, this microbial diversity introduces variability and challenges for process standardization and reproducibility. Understanding and managing community dynamics therefore represents a key frontier in balancing product authenticity with industrial consistency.

Despite their long history and technological maturity, fermentation-based processes face several challenges onat industrialon an industrial scale. Key limitations include product inhibition by organic acids and solvents, trade-offs between yield and productivity, high downstream processing costs, and variability associated with complex feedstocks. Scale-up from laboratory to industrial bioreactors further requires careful control of mass transfer, redox balance, and microbial stability. Addressing these challenges relies on integrated strategies combining strain improvement, process optimization, in situ product recovery, and techno-economic assessment to ensure competitiveness with petrochemical alternatives. Many of these challenges are fermentation-specific and were highlighted in the individual sections above [28,33,121]. 

Recent advances in metabolic engineering and synthetic biology are reshaping classical fermentation processes. Engineered *S. cerevisiae* strains with optimized redox balance, inhibitor tolerance, and expanded substrate spectra are increasingly applied in alcoholic and mixed fermentations. Specifically, systems biology tools such as genome-scale metabolic models, flux balance analysis, and multi-omics integration have enabled the identification of metabolic bottlenecks, redox imbalances, and by-product formation pathways, which can then be rationally targeted by metabolic engineering. In lactic and propionic fermentations, genome-scale metabolic models and CRISPR-based tools enable targeted modulation of acid yields, cofactor regeneration, and by-product formation. Similarly, engineered *Clostridium* species are being developed to improve solvent selectivity and butyrate productivity while reducing acid toxicity. These approaches, supported by multi-omics integration and adaptive laboratory evolution, transform traditional fermentations into controllable microbial cell factories [24,25,156].

Alcoholic fermentation underpins the production of fermented beverages, baked goods and bioethanol, linking cultural traditions with modern energy and chemical industries. Acetic fermentation is essential for vinegar and low-alcohol beverages and provides acetic acid as a versatile platform chemical for polymers, textiles, cosmetics and pharmaceuticals. Butyric fermentation is both a challenge in some fermented foods and a powerful biotechnological route to short-chain fatty acids, solvents and advanced biofuels. Lactic fermentation remains the backbone of fermented foods and functional/probiotic products and supplies lactic acid for biodegradable polymers and medical biomaterials. Propionic fermentation closes the circle by supporting cheese ripening, vitamin B_12_ production and a wide range of chemical, cosmetic and pharmaceutical applications.

Looking forward, several trends are likely to shape the future of fermentation-based technologies:

- Strain engineering and synthetic biology will enable the design of microbial cell factories with enhanced productivity, substrate flexibility and stress tolerance, including engineered LAB, *Saccharomyces*, *Zymomonas*, *Clostridium* and *Propionibacterium* strains.
- Multi-omics and systems biology approaches will deepen understanding of microbial consortia in spontaneous and mixed-culture fermentations, allowing rational manipulation of community structure and function to optimize product profiles.
- Valorization of agro-industrial by-products will expand, using fermentation to convert low-value streams into high-value compounds such as organic acids, biopolymers, aroma compounds and biofuels, supporting circular bioeconomy strategies.
- From a sustainability perspective, fermentation technologies increasingly support circular and zero-waste bioeconomy concepts. Alcoholic and lactic fermentations are widely applied to valorize agro-industrial by-products such as whey, molasses, fruit pomace, and cereal residues into ethanol, lactic acid, and functional metabolites. Acetic acid bacteria enable the upgrading of ethanol-rich side streams into vinegar and gluconic acid, while propionic and butyric fermentations convert lactate- or glycerol-rich wastes into preservatives, biofuels, and platform chemicals. Integrated biorefineries combining multiple fermentation steps exemplify how classical processes can be coupled to achieve near-complete carbon utilization, reduced waste generation, and lower environmental impact. Key challenges in transitioning toward sustainable bioprocessing include the heterogeneity and seasonal variability of waste streams, the presence of inhibitory compounds, logistical constraints in feedstock collection, and the need for robust strains capable of efficiently utilizing mixed and low-cost substrates.
- Development of functional and personalized foods will increasingly rely on tailored fermentations and starter cultures with targeted health effects, including modulation of the gut microbiota, immune system and metabolic homeostasis.

In parallel, technological innovation and digitalization are transforming fermentation monitoring and control. The integration of real-time sensing technologies for pH, redox potential, dissolved gases, and key metabolites, combined with soft sensors, digital twins, machine learning, and artificial intelligence-driven control systems, enables predictive modeling, early fault detection, and dynamic optimization of fermentation conditions. Such data-driven approaches are particularly valuable for large-scale and mixed-culture fermentations, where they support improved reproducibility, scalability, resource efficiency, and economic performance. Together with process intensification strategies such as membrane bioreactors, in situ product recovery, and high-pressure processing, these tools contribute to more sustainable, robust, and competitive fermentation-based bioprocesses [27,33,173]. 

## Figures and Tables

**Table 5:** Propionate-producing microorganisms, their primary substrates and fermentation products.

Genus/Species	Primary Substrate(s)	Main Fermentation Products	References
*Propionibacterium acidipropionici*	Glucose, sucrose, lactose, lactate, glycerol	Propionate, acetate, succinate, CO_2_	[111,144,189]
*Propionibacterium freudenreichii*	Lactate, glucose	Propionate, acetate, CO_2_	[111,144,189,190]
*Propionibacterium shermanii*	Lactate, glucose	Propionate, acetate, CO_2_	[111,144]
*Acidipropionibacterium thoenii*	Lactate, glucose	Propionate, acetate	[111,191]
*Acidipropionibacterium jensenii*	Lactate	Propionate, acetate	[111,191]
*Acidipropionibacterium microaerophilum*	Lactate	Propionate, acetate	[191]
*Clostridium propionicum*	Glycerol, lactate, alanine	Propionate, succinate, acetate, formate, n-propanol	[192,193]
*Clostridium neopropionicum*	Ethanol	Propionate, acetate	[121,193]
*Clostridium homopropionicum*	Glucose	Propionate	[121]
*Bacteroides fragilis*	Glucose	Acetate, propionate, lactate, succinate, formate	[194]
*Bacteroides ruminicola*	Glucose, cellobiose	Acetate, propionate	[194,195]
*Prevotella ruminicola*	Glucose, cellulose hydrolysates	Propionate, acetate, succinate	[195]
*Veillonella parvula*	Lactate	Propionate, acetate, CO_2_, H_2_	[196]
*Veillonella alcalescens*	Lactate	Propionate, acetate, CO_2_	[196]
*Selenomonas ruminantium*	Lactate, glucose	Propionate, lactate, acetate	[195,197]
*Selenomonas sputigena*	Glucose	Propionate, acetate	[197]
*Selenomonas lipolytica*	Glycerol, fatty acids	Propionate, acetate	[198]
*Megasphaera elsdenii*	Lactate	Acetate, propionate, butyrate	[148,150,199]
*Megasphaera micronuciformis*	Lactate	Propionate, butyrate, acetate	[148]
*Fusobacterium necrophorum*	Lactate	Acetate, propionate, butyrate	[150]
*Anaerovibrio lipolyticus*	Lipids, glycerol	Propionate, acetate, succinate	[200]

**Table 6:** Industrial applications of propionic fermentation and propionic acid across food, chemical, cosmetic, polymer and pharmaceutical sectors.

Application Area	Function/Role of Propionic Fermentation or Propionic Acid	Key Microorganisms/Compounds	Industrial or Health Relevance	Selected References
Dairy Industry (Cheese Ripening)	Conversion of lactic acid to propionic and acetic acids and CO_2_ during cheese maturation; contributes to flavor, aroma, and texture.	*Propionibacterium freudenreichii* subsp. *shermanii*, *P. acidipropionici*	Formation of characteristic eyes in Swiss-type cheeses (Emmental, Maasdam, Gruyère); development of nutty aroma; natural mold inhibition and extended shelf life.	[111,119,126,128,141,144,145,189,192]
Food Preservation	Natural preservative and antifungal agent; inhibits spoilage microorganisms and foodborne pathogens.	Propionic acid; sodium, calcium, potassium, and ammonium propionates	Preservation of bakery products, cheeses, and silage; suppression of *Aspergillus flavus*, *Bacillus* spp., *Salmonella* spp., *Listeria monocytogenes*; enhancement of product safety and shelf stability.	[98,103,106,107,109,110,111,119,165,189]
Biotechnological Production of Vitamin B_12_	Microbial biosynthesis of cobalamin (vitamin B_12_) through propionic fermentation.	*Propionibacterium freudenreichii*, *P. acidipropionici*	Used in pharmaceuticals and functional food fortification; essential for human metabolism.	[6,54,111,189,192]
Chemical Industry	Intermediate for synthesis of organic compounds, herbicides, and propionate esters.	Propionic acid and esters	Production of protective coatings, industrial varnishes, and solvents; valued for volatility, durability, and film-forming ability.	[14,47,75,111,112,119,189]
Construction and Cleaning Products	Antimicrobial additive in paints, adhesives, detergents, and impregnation agents.	Propionic acid and salts	Prevents microbial growth on surfaces, improving durability and hygiene in materials and environments.	[14,47,75,111,119,189]
Plastics and Polymer Industry	Intermediate for synthesis of cellulose propionate and biodegradable polymers.	Cellulose propionate; propionate esters	Used for films, packaging, optical materials, and bioplastic components; combines functionality with environmental sustainability.	[14,43,47,74,75,111,112,119]
Cosmetic Industry	Natural preservative and fragrance base; stabilizer in cosmetic emulsions.	Propionic acid and salts	Improves texture and stability of formulations; antimicrobial effect reduces synthetic preservative need.	[47,110,111,119,146,189]
Pharmaceutical and Veterinary Applications	Antibacterial and anti-inflammatory properties; therapeutic and auxiliary agent.	Sodium, calcium, and ammonium propionates	Used in treatment of skin infections, fungal diseases, and conjunctivitis; veterinary hygiene and hoof-care products.	[47,103,110,111,119,189]
Green and Circular Biotechnology	Integration of propionic acid production into sustainable bioprocesses and bioeconomy frameworks.	*Propionibacterium* spp.; bio-based propionate systems	Key biocomponent linking food, chemical, and pharmaceutical sectors; aligned with green chemistry and circular economy principles.	[6,9,14,33,50,75,111,119,165,189,192]

## Data Availability

No new data were created or analyzed in this study. The data presented in this study are available within the article.
